# Detecting continuous structural heterogeneity in single-molecule localization microscopy data

**DOI:** 10.1038/s41598-023-46488-z

**Published:** 2023-11-13

**Authors:** Sobhan Haghparast, Sjoerd Stallinga, Bernd Rieger

**Affiliations:** Department of Imaging Physics, Delft University of Technology, 2628 CJ Delft, The Netherlands

**Keywords:** Fluorescence imaging, Super-resolution microscopy, Image processing, Super-resolution microscopy

## Abstract

Fusion of multiple chemically identical complexes, so-called particles, in localization microscopy, can improve the signal-to-noise ratio and overcome under-labeling. To this end, structural homogeneity of the data must be assumed. Biological heterogeneity, however, could be present in the data originating from distinct conformational variations or (continuous) variations in particle shapes. We present a prior-knowledge-free method for detecting continuous structural variations with localization microscopy. Detecting this heterogeneity leads to more faithful fusions and reconstructions of the localization microscopy data as their heterogeneity is taken into account. In experimental datasets, we show the continuous variation of the height of DNA origami tetrahedrons imaged with 3D PAINT and of the radius of Nuclear Pore Complexes imaged in 2D with STORM. In simulation, we study the impact on the heterogeneity detection pipeline of Degree Of Labeling and of structural variations in the form of two independent modes.

## Introduction

Single-molecule localization microscopy (SMLM) is used to image biological samples at resolutions below the diffraction limit^1,2^. The resolution of SMLM is limited by the density of labeling and localization precision^3,4^. Fusion of multiple SMLM image datasets of chemically identical structures (particles) that are typically multi-component protein complexes with fixed spatial relationships, can improve the resolution as it increases the signal-to-noise ratio and overcomes under-labeling^5,6^. In SMLM the general idea is along the same line as in single particle averaging techniques (SPA) in cryo-EM^7,8^. Alignment of these particles is commonly achieved using either model-based registration methods^9,10^ or template-free registration methods^11–14^. Both approaches assume homogeneity of the dataset: the underlying structure is assumed to be the same for all particles. Structural heterogeneity, however, could be present in the data. These structural variations could originate from e.g. biological variations^6^ or from sample preparation^15^. In the field of cryo-electron microscopy (cryo-EM)^16–18^ methods to detect and cluster discrete variations are commonly used^19–22^. Recently, we have proposed a method to detect discrete structural variations in SMLM data with a clustering approach^23^.

The goal of the current paper is to develop a method that can detect continuous heterogeneity in specifically SMLM datasets. This offers the potential to study naturally occurring biological variations related to e.g. dynamics or development of protein assemblies in cells. Such a method also has the advantage that blurring of the final fusion result by the underlying continuous heterogeneity can be tackled by fusing parts of the dataset that are detected to be sufficiently homogeneous. The stated task of detecting continuous heterogeneity is challenging in view of statistical variations inherent to SMLM point datasets. Relevant factors for these statistical variations are the (anisotropic) localization uncertainty, which results in scatter of localizations around the fluorophore binding sites, repeated localizations corresponding to the same fluorophore, which leads to variations of the density of localizations, and underlabeling, which gives rise to randomly different subsets of the total set of binding sites that are actually labeled with a fluorophore. These effects make the image formation for SMLM essentially different from cryo-EM, and stand in the way of direct application of methods developed for cryo-EM, such as a recent proposal to investigate continuous heterogeneity using a deep learning-based mixed-dimensional Gaussian mixture model^24^. A learning-based approach for SMLM has been introduced recently^25^, but the classification tool needs manual annotation for training. Another statistical pattern recognition approach for classification (ECLiPSE^26^), requires segmentation, and therefore needs a high degree of labeling and signal-to-noise ratio. Approaches like LocMoFit (Localization Model Fit)^27^ can be seen as a way of fitting a point cloud with a parametrized geometric model built on a priori knowledge. This is in contrast to our approach where we use a data-driven analysis by extracting information without the use of prior knowledge. Model-based approaches^28^ in general are not ideal as they are susceptible to template bias and subjective model selection.

In this paper we present a model-free continuous heterogeneity detection method that works directly on localization coordinates in order to employ the full potential of the SMLM data. We apply our method to experimental data for the continuous detection of the height of DNA-origami tetrahedron structures imaged in 3D and of the radius of Nuclear Pore Complexes (NPCs) imaged in 2D.

## Methods

**Figure 1:**
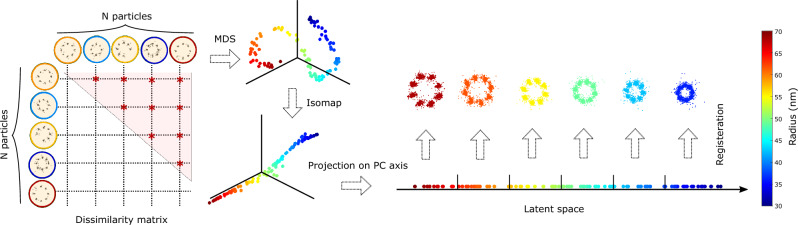
Continuous heterogeneity detection pipeline. N particles are registered using the all-to-all registration procedure resulting in $N(N-1)/2$ dissimilarity values. Multi-dimensional scaling embeds the dissimilarity matrix into an abstract Cartesian space (only the first 3 dimensions are shown). Points lying on a low-dimensional manifold are unrolled using Isomap. Projection of the unrolled manifold on the main principal component axis creates a 1D latent space in which the data is ordered based on the dissimilarity captured by the dissimilarity measure.

Our continuous heterogeneity detection (CHD) pipeline is illustrated in Fig. 1. It starts by computing a pairwise registration of all particles to obtain a dissimilarity matrix of the particles, similar to the approach of Huijben et al.^23^. The dissimilarity values between particles are mapped via multi-dimensional scaling^29^ to a high-dimensional feature space (MDS). This typically results in a low-dimensional manifold embedded in the high-dimensional MDS space. The low-dimensionality indicates that the particles are mostly alike but vary in one or just a few features. In the next step, the Isomap algorithm^30^ is used to “unroll” the data into a lower dimensional embedding. Finally, principal component analysis is used to project this representation onto the axis carrying the largest variation in a 1D latent space. This sequence of transformations preserves local ordering, and therefore, the ordering in 1D latent space carries the information on the continuous heterogeneity captured by the dissimilarity measure. Since neighboring particles in the latent space are structurally similar to each other, we can now divide them into bins and fuse the particles per bin.

In the following we describe the different steps of the algorithm in detail.

### Pairwise registration

We use the all-to-all registration^11^ to register *N* particles from a 2D/3D SMLM dataset. Each particle is independently registered to all other particles in the dataset using a combination of Gaussian Mixture Model (GMM) registration^31^ and the Bhattacharya cost function^14^. First, particles are aligned using GMM registration with multiple initial poses, which results in a set of rotation matrices and translation vectors. The final registration parameters are those that maximize the Bhattacharya cost function over the GMM optima for the different initializations. This procedure results in transformation parameters (rotation and translation) and the optimum cost value for each pair. The width of the Gaussian distribution (scale parameter) used in GMM registration is obtained by registering ten random groups of particles for different scales. In this scale-sweep approach, the scale parameter which gives the highest Bhattacharya cost value for all test sets is chosen as the scale value to be used for the whole dataset. It is necessary to select the proper scale value to avoid blurring of nearby binding sites or overfitting on each localization. The elements of the $N\times N$ matrix of optimum cost values (or rather the upper triangular part of this matrix) are normalized by the number of localizations for the two corresponding particles^23^. This makes the cost function matrix less sensitive to variations in the number of localizations. Finally, all pairwise cost values (quantifying the similarity between pairs of particles) are subtracted from the maximum pairwise cost value to create a dissimilarity matrix.

### Multi-dimensional scaling (MDS) space

We assign coordinates to the *N* particles in a high-dimensional (dimension $D=30$) space, by interpreting the values of the dissimilarity matrix as the Euclidean distance in this high-dimensional space, the so-called MDS space. This is done by iteratively updating the coordinates in MDS space to minimize the stress function:

1 $$\begin{aligned} S = { {{\sum \nolimits _{ij} {{{({d_{ij}} - ||{x_i} - {x_j}||)}^2}} }} }, \end{aligned}$$

where $d_{ij}$ is the pairwise dissimilarity between particle *i* and *j*, and $x_{i}$ is the MDS position vector of particle *i* ($i=1,2,\ldots N$). We found that a dimensionality $D>15$ resulted in a value for the stress function smaller than $10^{-4}$, which was sufficient for all our applications. As a rule of thumb, the number of dimensions is set to be $D=30$^23^.

### Isomap

We have observed that the point cloud in MDS space can and often is distributed across a lower-dimensional manifold. In addition, the ordering of the points on this manifold correlates with the ordering from the dissimilarity metric. In order to take advantage of this in case it occurs, we use a global geometric framework for nonlinear dimensionality reduction, the so-called Isomap algorithm^30^. The Isomap algorithm is used to unroll the low-dimensional manifold embedded in the high-dimensional MDS space by flattening the curved manifold, preferably into a non-curved shape. This in turn enables easier detection of the dominant directions of variation in the dissimilarity measure across the data in MDS space. Isomap unrolls the manifold while keeping the number of dimensions the same as the original MDS space using the following steps: (1) Clustering the particles^32^ to find the *k* nearest neighbors based on the Euclidean distance for all *N* particles in MDS space. (2) Connecting the *k* neighborhoods for each point to construct a proximity graph. (3) Computing the shortest pairwise distance for all pairs of points in the graph. This results in a new $N\times N$ matrix in which the elements represent the geodesic distance. (4) Embedding the geodesic distance matrix into MDS space by minimizing the stress function, keeping the number of dimensions the same. The parameter *k*, which corresponds to the number of neighbors used in Isomap, should be empirically chosen based on the distribution of the particles in MDS space (typically between 4 and 12). We empirically found $k=4$ works well. If *k* is too high, unrolling cannot be performed properly as neighbors can then be found not just along the manifold, but also via a “shortcut” through empty space. In cases where the successive differences between explained variance on the first three axes are higher than half of the average variance explained, Isomap can reveal the low dimensional latent space. Otherwise, it can not further reduce the number of dimensions due to an isotropic distribution in MDS space.

### Principal component analysis (PCA)

After unrolling the data in the high dimensional MDS space, we used PCA to identify the direction of the largest variation. Here the axis with maximum variance explained corresponding to the highest eigenvalue was selected as the one embedding the maximum mode of variation in the data measured by the Bhattacharya cost function. Subsequently, by projecting all particles on its main principal component axis, a 1D latent space is created, which corresponds to some of the continuous heterogeneity that exists in the dataset.

### Reconstruction per bin

To visualize the continuous heterogeneity revealed in the latent space, particles are divided into bins, and a single reconstruction for each bin is made. The way in which the particles are distributed over the bins is adapted to the distribution of the particles in the latent space. For a (near) uniform distribution of particles, the bin width is set such that all bins cover the dynamic range of values in latent space. For a more bell-shaped distribution of particles, the bin width is set to be equal to half of the fitted standard deviation of the distribution. The total number of bins is typically chosen in the range of 5–10, but can in principle be chosen arbitrarily, provided there are more than approximately 10 particles per bin. The resulting reconstructions per bin are expected to be more faithful since the particles in each bin are structurally close to each other.

### Particle fusion

The superparticle reconstructions per bin are made with a template-free method developed previously by us^13^, based on earlier work by Evangelides and Horaud^33^, the so-called Joint Registration of Multiple Point Clouds (JRMPC).

### Model-based shape parameter estimation

We identify the coordinate axis in 1D latent space indicating the heterogeneity parameter. In an experimental 2D NPC dataset, we compare the latent space coordinate to the estimated radius of the ring structure. To estimate the radius, we center all particles by subtracting the mean of all localizations in a particle from the localizations, as in Heydarian et al.^11^. Subsequently, the localization coordinates are transformed into polar coordinates. We take the mean of the radial coordinate as an estimation of NPC radius. To estimate the precision of the model we calculate the FWHM of the radius histogram and divide it by the square root of the number of localization events to find the standard error of the mean. In an experimental 3D DNA-origami tetrahedron dataset, we compare the latent space coordinate to the estimated height of the tetrahedron structure. To this end, we manually align the particles in each bin along the *z*-axis and project all localizations on the *z*-axis, giving a histogram of *z* coordinates of all localizations in the particle. This histogram has two peaks, one corresponding to the three binding sites in the base plate of the tetrahedron, and the other corresponding to the tip of the tetrahedron. By fitting a mixture of two Gaussian distributions to this histogram, we find the height as the difference between the mean of the two fitted Gaussian distributions. In a simulation study on elliptically shaped 2D NPCs we estimate the ellipticity of the ring structure as in Huijben et al.^23^ by finding the center of the 8 blobs in each particle using *k* means clustering, followed by fitting an ellipse to the centers of the 8 blobs.

### Data acquisition of 2D NPC

Experimental NPC data was acquired with the following protocol. U2OS Nup96-SNAP cells (Cell Lines Services, from Jan Ellenberg, EMBL) were seeded on collagen-coated 8-well chambers slide (1.5NA, LabTek II #155409) with 3 $\upmu$g/mL aphidicolin (Millipore #178273-1MG) and incubated overnight at 37°C. Cells were then pre-fixed with 2% Paraformaldehyde (PFA, Electron Microscopy Sciences #1570-S) for 30 sec, permeabilized with 0.1% Digitonin (RPI #43065-0.1 ) for 30 min and additionally fixed with 2% PFA for 10 min. PFA was quenched with 20 mM Tris pH 8.0 (G-Biosciences #R002) for 5min. Samples were then blocked with 10% Fetal Bovine Serum (FBS, Hyclone #SV30014.03) for 30 min and then stained with 500 nM AlexaFluor647-BG for SNAP-tag (NewEngland Biolabs #S9136S) with 1 $\mu$M of Dithiothreitol (DTT, American Bioanalytical #AB00490) in 10% FBS for 1 h at room temperature. Samples were prepared for STORM imaging with 100 mM Cysteamine Hydrochloride (MEA, Sigma #M6500-25G), 1% GLOX oxygen scavenger buffer (40 $\mu$g/mL catalase (Sigma #C40-100MG) and 500 $\mu$g/mL glucose oxidase (Sigma #G2133-50KU)) and a second buffer (10 mM NaCl, 50 mM Tris pH 8 and 10% Glucose). Images were taken using an Oxford Nanoimager (ONi) STORM microscope with oil-immersion objective (100X, 1.4NA) and laser power density of $4~kW/cm^2$. For imaging acquisition, a 641 nm laser was used to excite AF647-BG and a 405 nm laser was used to enhance blinking. Samples were pre-bleached with 190 mW of 641 nm laser (1500 frames), then acquisition was done with 120 mW of the 641 nm laser (5000 frames) and enhanced blinking with 0.2 mW of 405 nm and 120 mW of 641 nm (5000 frames).

## Results

We applied the proposed CHD analysis pipeline to two experimental datasets, a 3D DNA origami tetrahedron dataset, and a 2D NPC dataset. We also investigated limitations of the method by two simulation studies, on the impact of the Degree Of Labeling (DOL) and on the sensitivity to two independent modes of continuous variation.

### Continuous distribution of 3D DNA-origami tetrahedron height

**Figure 2:**
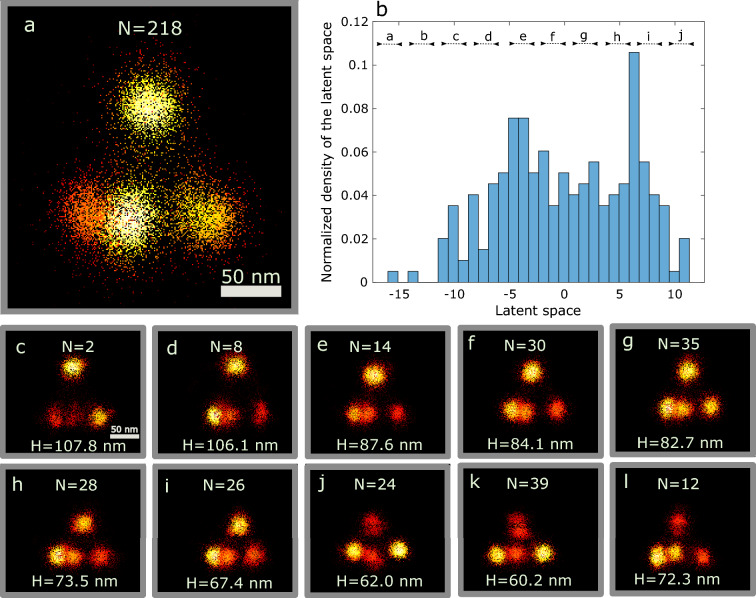
Continuous distribution of DNA origami tetrahedron height. (**a**) All particles are registered using fast particle fusion based on the homogeneity assumption. The fusion of structurally different particles causes blurry and elongated blobs. (**b**) The distribution of the particles in latent space. (**c–l**) Fusion of particles in 10 bins sorted along the latent space coordinate, showing a clear variation in the height of tetrahedrons. Scale bar in (**c**) applies to (**d–l**) as well. Colormap represents the localization density.

We applied our CHD pipeline to a three-dimensional DNA-origami tetrahedron data set, imaged with DNA-PAINT^23,34^. This data set consists of 218 particles with an edge length of around 100 nm and a height of around 90 nm. There is a variation in tetrahedron height, which was analyzed previously by Huijben et al.^23^ with their clustering approach. We have applied our CHD method to this data set as well and the key results are shown in Fig. 2. The reconstruction of the whole data set (Fig. 2a) was obtained using fast particle fusion based on JRMPC^13^. Figure 2b shows the distribution of particles over the latent space coordinate. There is a clear correlation of the found latent space coordinate with a continuous height variation of about 45 nm in the entire data set, providing additional support for the validity of our method. The variance explained in the first PC axis is 23% (clearly larger than in the next directions, which give rise to 14, 9, 5, and 4% variance explained). We divided the latent space into 10 bins with equal length in latent space as the particles are almost uniformly distributed in the latent space. Figure 2c–h shows the reconstructions for each bin. These reconstructions have less elongated blobs than the overall reconstruction of Fig. 2a. A 3D reconstruction of each bin can be seen in Supplementary video 1.

### Continuous distribution of 2D NPC radius

The NPC dataset consists of 1339 particles imaged with 2D STORM (see Methods section). Each particle was picked (cropped) manually from a single SMLM image. The shape of lower-dimensional projections of the distribution of points in MDS space turns out to be closer to an ellipsoid structure as opposed to a more flat shape. As a result, the Isomap step does not provide additional value in this case, and we directly applied PCA to the distribution in MDS space. We attribute the more diffuse topology of the manifold in MDS space to other modes of variation in addition to the dominant one (NPC radius). These confounding modes of variation could be related to other modes of structural variation, and SMLM specific statistical variations such as Degree Of Labeling (DOL).

**Figure 3:**
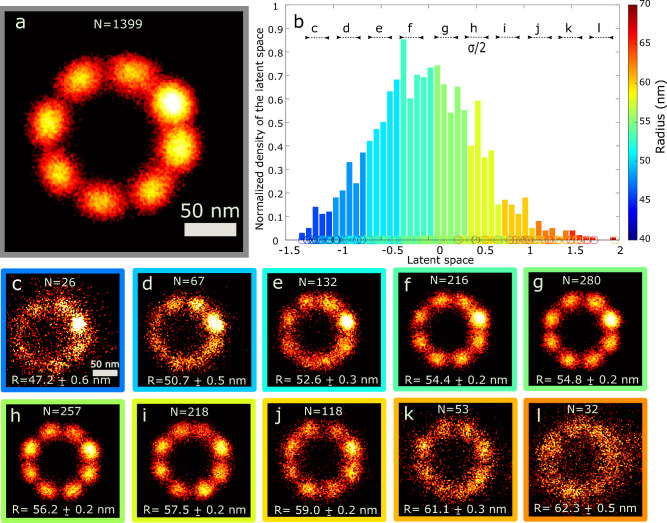
Continuous distribution of 2D NPC radius. (**a**) Fusion of 1399 NPCs imaged with STORM using fast particle fusion with homogeneity assumption. (**b**) The normalized histogram of the latent space is fitted by a Gaussian distribution, and the size of each bin is selected as half of the fitted standard deviation. Images (**c–l**) correspond to registered particles inside each division using particle fusion^13^. Scale bar in (**c**) Applies to (**d–l**).

By estimating the size of each particle as explained in the Model-based shape parameter estimation in Method section, we observed a continuous variation in the radius of the scaffold that seems drawn from a Gaussian distribution with a mean of around 55 nm, and a standard deviation of around 4 nm. This continuous heterogeneity causes blurriness in the reconstruction of all particles in the dataset (Fig. 3a) that assumes homogeneity of the underlying data. Fig. 3b shows the results of analyzing this dataset with our CHD algorithm. There is a very clear correlation between the latent space coordinate and the independently assessed radius, validating our method for discovery of continuous heterogeneity without any prior assumptions. Although the first principal axis shows a very good correlation with radius, the variance explained in this axis is only 4%. This may be related to the more diffuse topology of the MDS manifold compared to the 3D DNA-origami tetrahedron case. We also inspected the second or higher principal axis but did not observe a correlation to another structural feature.

**Figure 4:**
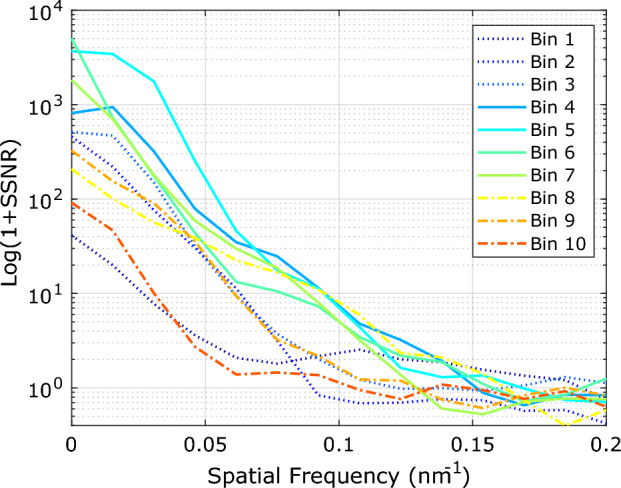
Spectral signal to noise curves for each division. This figure illustrates that bins 4–7 have higher SSNR than bins 1–3 and 8–10, which confirms the better reconstruction quality of the middle bins.

Figure 3c–l shows reconstructions of particles in 10 bins, defined according to the observed bell-shaped distribution of the latent space coordinate (see Methods section). The results indicate that the registration of particles in the middle bins (Fig. 3f–i) leads to better reconstructions compared to the first (Fig. 3c, e) and last bins (Fig. 3j–l). As a quantitative measure, we calculated the spectral signal to noise^35^ (SSNR) curves for each bin. To that end we divided each bin into two halves and registered each part independently using the fast particle fusion approach. After alignment of the two halves, we applied a random rotation in view of the 8-fold rotational symmetry of the structure, thereby avoiding hotspots. Figure 4 shows that for the middle latent space bins (4–7), the SSNR curves are higher than for the first (1–3) and last (8–10) bins, in agreement with the visual quality of the reconstructions per bin in (Fig. 3c–l). All SSNR curves level off to a noise plateau for spatial frequencies higher than approximately 0.15 nm$^{-1}$, indicating that the smallest features in the reconstructions are about 6 nm.

**Figure 5:**
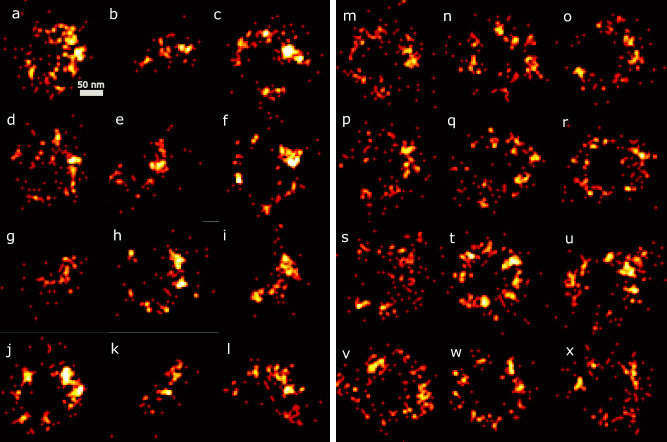
Outliers detected by the CHD pipeline. Images (**a–l**) correspond to particles that are in low-density regions of the latent space. As reference, images (**m–x**) are randomly selected from the whole data set.

The reason for the relatively poor reconstruction of the extreme bins is not just the lower number of similar particles that contribute to the reconstruction, but also that these bins are contaminated with outlier particles. This suggests an additional value of our proposed method, namely outlier particle detection. Figure 5 shows examples of particles that can be designated as outlier particles compared to randomly selected valid particles. As threshold for the definition of outlier, we take the extreme 1% of the distribution of particles in latent space. Visually, these outlier particles are relatively remote from the expected ring shaped point clouds.

### Simulation on impact of DOL

**Figure 6:**
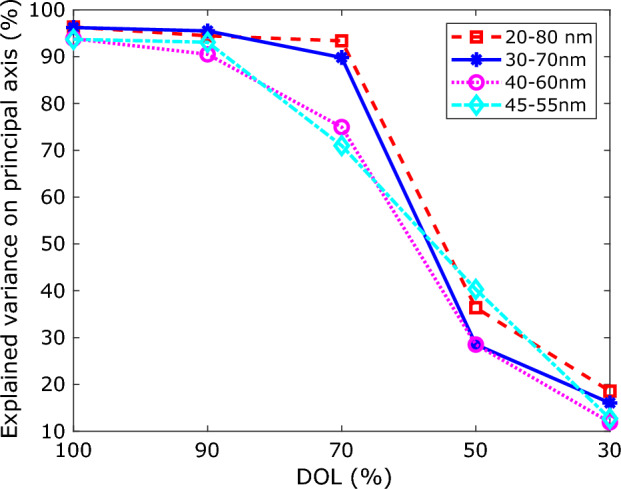
Performance of continuous heterogeneity detection with DOL. Each curve consists of 5 data points, pertaining to datasets with five DOL values (30, 50, 70, 90, 100%). The radius of the particles is drawn from a uniform distribution with ranges indicated in the legend.

A low Degree Of Labeling (DOL) of binding sites is a common problem in SMLM. It can be expected that DOL also has an impact on the ability to detect continuous heterogeneity in a dataset, as the randomness of which binding sites are labeled (and which not) affects the imaged structure of each individual particle. We investigated the impact of DOL by a simulation study of a 2D NPC structure. We applied our CHD pipeline to simulated data sets with five DOL values (30, 50, 70, 90, and 100%), and for particle radii drawn from uniform distributions with four ranges. The simulation is performed as Huijben et al.^23^ with 250 particles in each dataset. Figure 6 shows the variance explained on the first PC axis (the latent space coordinate) as a function of DOL for the four distributions. As expected the variance explained decreases with decreasing DOL. If we take 50% as a minimum value for this performance criterion, we can conclude that the DOL should be at least in the range 50–70%.

### Simulation on two modes of variation

**Figure 7:**
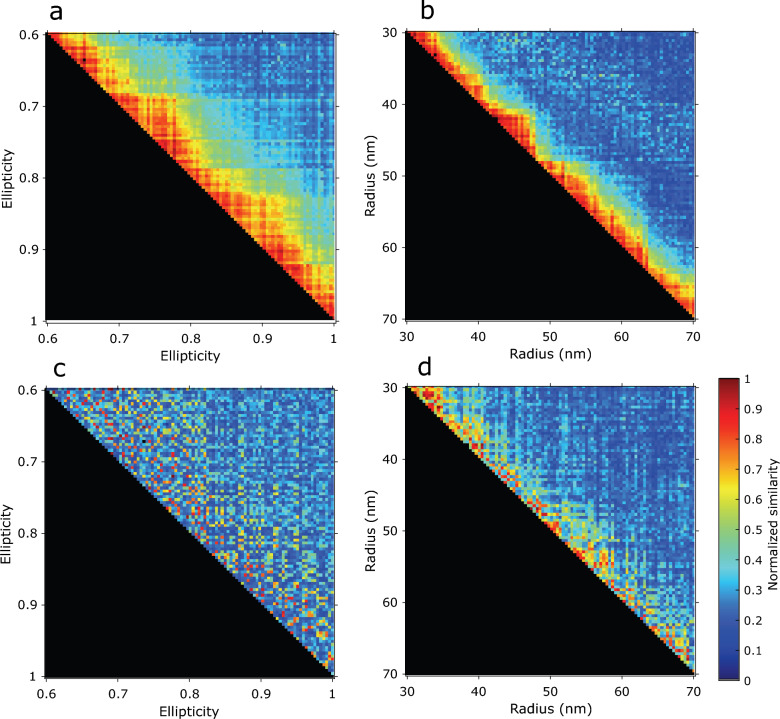
Sensitivity analysis of the cost function to two modes of variation. Plots illustrating the normalized pairwise cost value (similarity measure) for a variation in radius and/or ellipticity of simulated 2D NPC. (**a**) Cost values of a dataset with uniform distribution over ellipticity ratio ranging from 0.6 to 1. Particles are sorted based on their ellipticity from top to down and left to right in the matrix. The values in each column show the sensitivity of a particle with specific ellipticity to the other particles. As a result, the color separation over the diagonal band refers to the sensitivity of the method to the variations in ellipticity. (**b**) Cost values of a dataset with uniform distribution over the radius ranging from 30 nm to 70 nm. (**c, d**) Cost values of a dataset with two modes of variations. In (**c**) particles are sorted based on their ellipticity, while in (**d**) sorting is based on radius. A comparison of the diagonal bands in (**c**) and (**d**) shows that the method is more sensitive to variations in radius than in ellipticity.

So far, we have only considered cases in which there was a single, dominant mode of variation. To evaluate the ability of the CHD method to detect two modes of variation simultaneously, we made a simulation study of 2D NPC particles with both a variation in radius (uniform distribution ranging from 30 to 70 nm) and a variation in ellipticity of the ring (uniform distribution ranging from 0.6 to 1.0). The outcome was compared to simulations with only a variation in radius or in ellipticity. Figure 7 shows the similarity matrices for these different simulations. The matrices for single-mode variation simulations (Fig. 7a, b) were ordered such that the particles are sorted based on the ellipticity or radius of the particles. As a consequence, by moving from left to right in each row in Fig. 7a, b, the similarity value decreases since each particle is compared with a less similar structure in terms of ellipticity or radius. This applies to every row in the matrix and creates a diagonal band of higher similarity values. The width and the average hue of this diagonal band in the matrix are related to the sensitivity of the similarity measure to the ellipticity or radius. If we apply the same particle ordering procedure for either the ellipticity or the radius for two-mode variation simulation (Fig. 7c, d), it turns out that the width and hue of the diagonal band is limited or seemingly absent. This indicates that the single similarity metric we use might have difficulties to provide sufficient information to detect multiple modes of variation.

**Figure 8:**
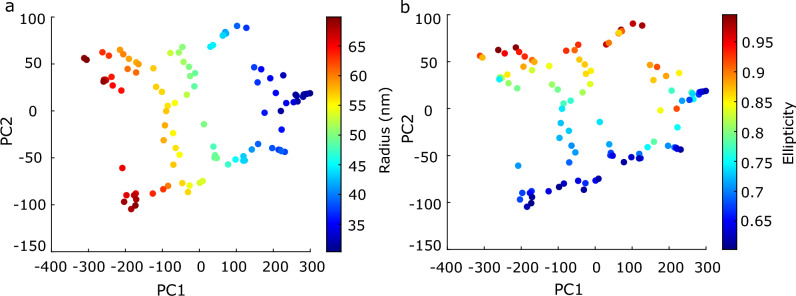
2D Latent space for two modes of variation. (**a**) Distribution of particles in the 2D latent space color coded respectively with the ground truth radius. (**b**) Distribution of particles in the 2D latent space color coded respectively with ellipticity.

In the simulated dataset with two modes of variation it appeared nevertheless possible to detect both modes. Figure 8 shows the distribution of particles in the 2D latent space (first and the second principal axes), where the color code shows the radius and ellipticity ground truth, respectively. Clearly, the variation in radius corresponds with the first principal axis, while the variation in ellipticity corresponds with the second principal axis. While these initial simulation results are encouraging, we have not been able to detect multiple modes of variation in both experimental datasets we studied. Several confounding factors, in particular a stronger underlabeling, could be limiting in detecting multiple variation modes in experiment.

## Discussion

In summary, we have developed a model-free continuous structural heterogeneity tool to sort particles based on a dissimilarity measure. We successfully detected continuous structural variation in different localization microscopy datasets, such as DNA-origami tetrahedrons and NPCs, which led to more faithful fusions.

The method should be applicable to any SMLM dataset that consists of particles that share a similar structure but vary in conformational state. In cryo-EM studying structure variation is applied to e.g. variation of position or motion of side groups. Similar variations could potentially be visible in our approach albeit at a lower resolution due to the imaging modality.

It is not clear how many samples are required per bin to detect structural heterogeneity, let alone how this number of samples depends on localisation precision and degree of labelling. Already for normal averaging, with the assumption of structural homegeneity, it is unclear how the FRC resolution of the reconstruction depends on the number of particles. This is very different from cryo-EM SPA where each extra particle reduces the noise and the improvement follows the expected $1/\sqrt{N}$ scaling). As best practice, we inspect the result and then judge if the numbers have been sufficient.

We found several limitations of the proposed template-free continuous heterogeneity detection method. Firstly, it is not a priori clear if the Isomap unrolling step is of use or not. Secondly, picking up modes of variation like variations in DOL, that have no clear geometrical interpretation such as size parameters, turn out in simulation to be too challenging to detect. Thirdly, the detection of multiple continuous modes of shape variation in SMLM data remains unsolved, despite initially hopeful simulation results. This may be due to the poorer quality of experimental data and variation modes that are entangled differently than foreseen in simulation. Finally, the method is based on a single dissimilarity metric that may be expected to have different sensitivities to different modes of variation.

An alternative to the proposed method may be to fit an a priori model to the data and then sort the data by the distribution of model parameters obtained by the fit. A major drawback of such an approach, however, is that the outcome of the analysis is prone to biases induced by the assumed model. A more fruitful next step may be to consider multiple metrics that quantify specific features and/or particle similarity for sorting the particles. This can be performed either by a semi-template-free approach with multiple features that are designed to be sensitive to specific modes of variation or by generating sets of more abstract features using deep neural networks and auto-encoders^36^.

### Supplementary Information

Supplementary Information.

## Data Availability

Data and codes are publicly available. Updated versions of the software can be downloaded from https://gitlab.tudelft.nl/imphys/ci/chd. The single molecule localization data is accessible via 4TU.research repository at https://data.4tu.nl/private_datasets/ML40deqg5qxTOaLstirZjiuZg82GLELrO9FSK-qzB0s.
