# Supplementary figures and images for: Detecting continuous structural heterogeneity in single-molecule localization microscopy data
Source: Sci Rep. 2023 Nov 13;13:19800. doi: 10.1038/s41598-023-46488-z (PMC10643625; doi:10.1038/s41598-023-46488-z)

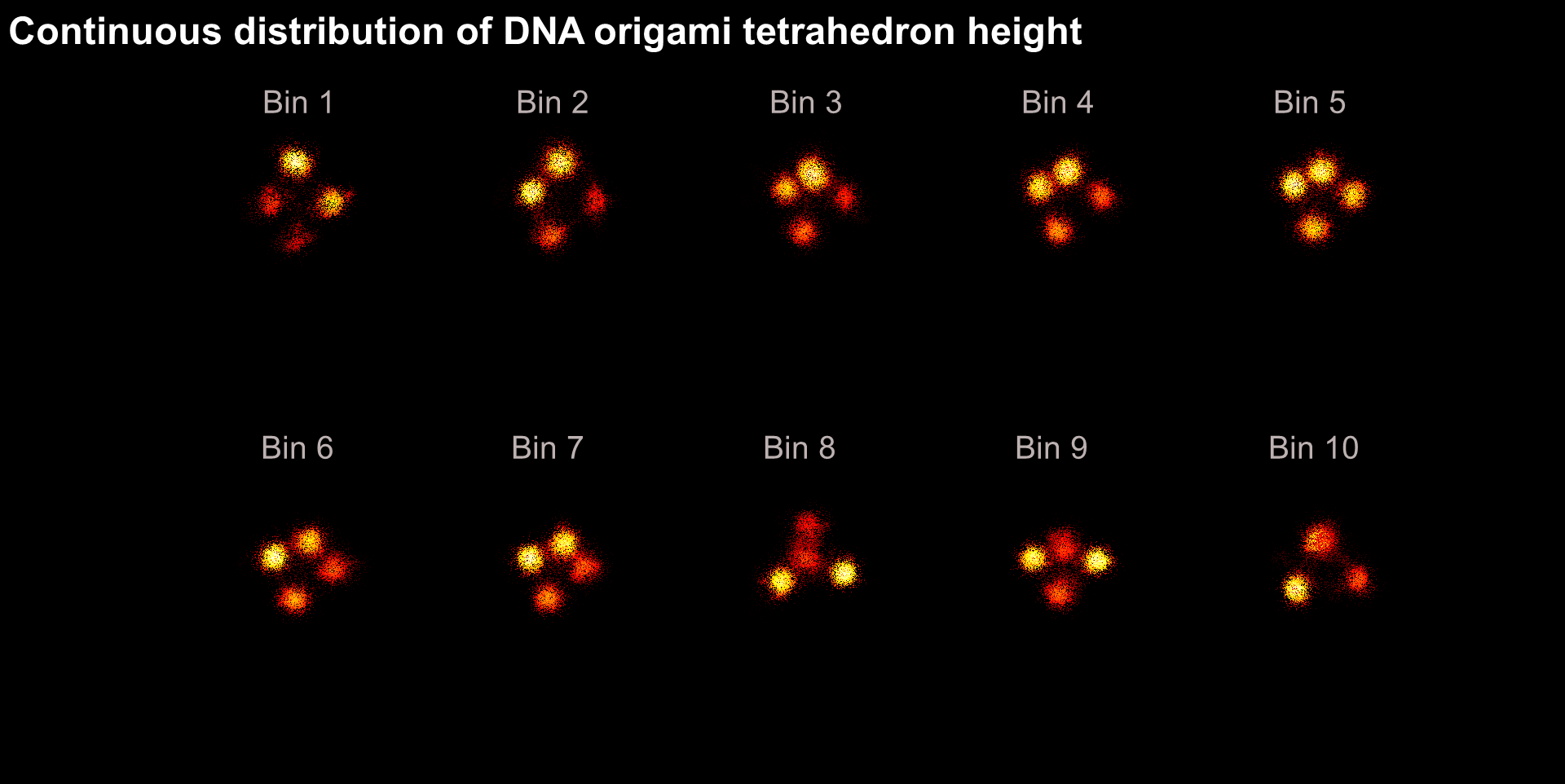

Supplement: Supplementary file 1 — Supplementary Information. [file 41598_2023_46488_MOESM1_ESM.gif]
